# Can we predict the direction of sudden shifts in symptoms? Transdiagnostic implications from a complex systems perspective on psychopathology

**DOI:** 10.1017/S0033291718002064

**Published:** 2018-08-22

**Authors:** Marieke Wichers, Marieke J. Schreuder, Rutger Goekoop, Robin N. Groen

**Affiliations:** 1 University of Groningen, University Medical Center Groningen, Department of Psychiatrie, Interdisciplinary Center Psychopathology and Emotion Regulation (ICPE), Groningen, The Netherlands; 2 Department of Mood Disorders, Parnassia Group, PsyQ, The Hague, The Netherlands

**Keywords:** Complex systems, psychopathology, transdiagnostic approach

## Abstract

Recently, there has been renewed interest in the application of assumptions from complex systems theory in the field of psychopathology. One assumption, with high clinical relevance, is that sudden transitions in symptoms may be anticipated by rising instability in the system, which can be detected with early warning signals (EWS). Empirical studies support the idea that this principle also applies to the field of psychopathology. The current manuscript discusses whether assumptions from complex systems theory can additionally be informative with respect to the specific symptom dimension in which such a transition will occur (e.g. whether a transition towards anxious, depressive or manic symptoms is most likely). From a complex systems perspective, both EWS measured in single symptom dynamics and network symptom dynamics at large are hypothesized to provide clues regarding the direction of the transition. Challenging research designs are needed to provide empirical validation of these hypotheses. These designs should be able to follow sudden transitions ‘live’ using frequent observations of symptoms within individuals and apply a transdiagnostic approach to psychopathology. If the assumptions proposed are supported by empirical studies then this will signify a large improvement in the possibility for personalized estimations of the course of psychiatric symptoms. Such information can be extremely useful for early intervention strategies aimed at preventing specific psychiatric problems.

## Introduction

### Sudden transitions in symptom levels

**Fig. 1.**
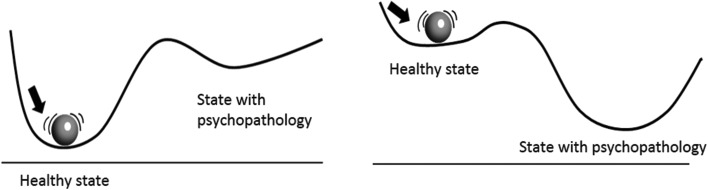
Two dimensional stability landscape. The ball represents the current state of a complex system and the line constitutes a surface showing the stability of the ball (or system state) in the current situation. In the left panel, the state of the system is in a deep basin of attraction, meaning that it will take a considerable perturbation of the system before psychopathology can develop. In the right panel, however, the situation is different. Here, only a small perturbation may already be enough for a transition towards a state of psychopathology.

In the past years, there has been renewed interest in the potential application of complex systems theory in the field of psychiatry (Heinzel *et al*., 2014; Borsboom, 2017; Haken and Tschacher, 2017; Nelson *et al*., 2017; Schiepek *et al*., 2017). In short, complex system theory entails that complex systems, ranging from ocean ecosystems to climate, financial markets or the evolutionary development of species, all have certain principles in common that predict their behaviour. These relate, for example, to the resilience of a system to remain in its present stable state. High resilience refers to a high level of stability of the system (deep basin of attraction) meaning that the system can easily face perturbations without being tipped out of its current equilibrium (Scheffer, 2009) (see Fig. 1).

In complex systems, this level of resilience may slowly diminish, even without noticeable signs. Once low, the system is highly instable and at this point even very minor contextual disturbances, also called perturbations, can push the system over a tipping point towards another basin of attraction (Scheffer, 2009). This is why complex systems are characterized by sudden transitions, so-called phase transitions, that appear to emerge ‘out of the blue’. Similar transitions have been observed in psychiatry, as psychiatric symptoms sometimes (re)appear in a very abrupt way (Hayes *et al*., 2007). Already at the end of the previous century the idea arose, that psychological phenomena might also behave according to the principles of complex systems (van der Maas and Molenaar, 1992; Hayes and Strauss, 1998; Beirle and Schiepek, 2002; Schiepek and Perlitz, 2009). If this is indeed the case, this is very relevant since the principles of complex systems teach us important things about the nature of psychopathology and how to understand and foresee sudden transitions in symptoms. One of the interesting consequences would be that the *early identification* of alterations in the level of instability of the system could reveal the proximity of the system's tipping point, or in other words, the likelihood that a sudden shift in symptoms occurs (Scheffer *et al*., 2009, 2012). Some recently conducted studies were able to translate this idea into simulation studies and empirical designs that attempted to test this assumption in the field of psychiatry (Schiepek *et al*., 2009; van de Leemput *et al*., 2014; Cramer *et al*., 2016; Wichers *et al*., 2016). Most of the focus in these studies has been on the possibility of foreseeing shifts in the levels of symptoms (increasing or decreasing levels). Yet, it would be important to not only foresee a shift in symptom level, but to also foresee the type of symptoms in which the transition occurs. For example, we want to foresee, if someone is approaching a symptom transition, whether that is a transition characterized by increasing manic, anxious or depressive symptoms. In this paper, we therefore want to explore whether we can extend the assumptions based on a complex systems perspective on psychopathology to foresee the *type* of these symptom shifts. Thus, rather than applying assumptions from the complex systems perspective on foreseeing level shifts in symptoms on a single dimension of psychopathology (in which the constituent symptoms can move from being absent to being present), it would be interesting to explore whether we could extend the impact of these assumptions to a multidimensional psychopathological space, in which we can foresee *what types of symptoms* are likely to develop.

The ability to detect the direction of transitions in psychopathology in high-risk individuals is highly relevant. During subclinical stages of psychopathology, people often experience a combination of symptoms that cross a wide range of psychopathological dimensions (Fusar-Poli *et al*., 2014; McGorry and Nelson, 2016). Therefore, it is often entirely unclear how and towards what type of symptoms psychopathology will develop in these high-risk individuals. This, however, is an urgent question as optimal clinical decision making in an early phase is important to reduce and prevent further development of psychopathology (McGorry *et al*., 2006; Cross *et al*., 2014). This urgency is expressed in the amount of resources that are invested in the development of adequate clinical staging and profiling techniques (Wigman *et al*., 2013; McGorry *et al*., 2014; Berk *et al*., 2017). The complex systems perspective may contribute to these aims as it provides a complementary angle from which to understand the development of psychopathology and find solutions to improve personalized prediction.

This manuscript will provide an overview of ideas and hypotheses that follow from taking a complex systems perspective on psychopathology, focusing on foreseeing the type of symptoms that are most likely to show sudden transitions. First, we will describe in more detail what parts of complex systems theory, regarding early warning signals (EWS) and transitions, have already been related to the field of psychopathology. Second, we will explain what additional predictions may follow from this theory with respect to foreseeing and differentiating the type of sudden shifts in symptoms. Also, we will discuss what research designs would be needed to empirically test these predictions and the relevance of these ideas for clinical practice.

### Support for a complex systems perspective on psychopathology

As mentioned above, there are reasons to assume that psychopathology behaves according to the principles of complex systems. First, sudden shifts in symptoms are observed in patients. Although psychopathology seems dimensional in nature in the sense that individuals can be anywhere on a continuum between having no symptoms and having severe symptoms, the road of symptom change within individuals can be much bumpier. Patients often report sudden relapses or sudden improvements in symptoms (Hayes *et al*., 2007; Tang *et al*., 2007; Heinzel *et al*., 2014). This has been confirmed by statistical analyses of symptom patterns over time in depressed patients, which revealed that most patients show a bimodal distribution in symptoms. In other words, they experience either low levels or high levels of symptoms (Hosenfeld *et al*., 2015). This suggests that they experience sudden jumps in their symptom levels. Also, a recent (*n* = 1) double-blind time-series experiment (Wichers *et al*., 2016), in which levels of symptoms were weekly and prospectively monitored over 239 days, confirmed the presence of a sudden jump in depressive symptoms in this person. At this change point, the level of symptoms suddenly went up and seemed to stabilize afterwards at a higher point on the continuum of depression. Although not all symptom transitions may occur in an abrupt fashion, these observations at least suggest that abrupt symptom changes are quite common, which is in line with the expectations from complex system theory. Currently, extensive empirical research that has mapped symptom patterns in patients frequently and prospectively is lacking. More research is thus needed to confirm the assumption that symptom transitions often occur in an abrupt fashion.

Second, verbal descriptions of patients suggest that sudden and discontinuous changes in their symptom experience (Hayes *et al*., 2007) may occur in the absence of an obvious, temporally proximal cause or reason. From a traditional approach, these unexpected symptom changes are difficult to explain as we know that external causes play an important role in symptom development. Logic dictates that changes in symptoms are directly preceded by changes in specific factors (such as in the social environment, stressful events or therapy). From a complex systems perspective, however, unexpected symptom change does make sense. This theory proposes that large shifts can also occur following minor, seemingly innocent stressors (Boeing, 2016). These shifts are most likely when a system's resilience to remain in its current basin of attraction is very low (Fig. 1), meaning that the system is in an unstable situation. From a complex systems perspective, such an unstable situation may result from the impact of a distal cause that happened some time ago, and that gradually led to the present loss of resilience. When resilience becomes very low, even minor seemingly unimportant disturbances can tip over the system to an alternative state (see Fig. 1). This can explain why patients may experience long intervals between potential environmental causes and the onset of their symptoms. Also, it can explain why sudden shifts in symptoms may occur in the absence of any obvious immediate trigger. Later we discuss how stability of the system can be empirically assessed.

Third, elements within complex systems are in a continuous and complex interplay with each other. In many complex systems, reinforcing feedbacks are present that, if strong enough, can push the system to another alternative state (Scheffer, 2009). Such feedback loops are also likely to occur between mental states. Recent studies have confirmed the presence of feedback loops through network models, which showed that negative mental states, such as feeling down or irritated, are related to the occurrences of other negative mental states later in time. These effects may form vicious circles (Wichers, 2014). Findings from most studies supported the hypothesis that reinforcing feedback loops were more pronounced in people with either higher levels of psychopathology or at risk of psychopathology, compared with individuals in the general population (Pe *et al*., 2015; Wigman *et al*., 2015; Bringmann *et al*., 2016; Klippel *et al*., 2017), although this was not confirmed by all studies (Eijlander *et al*., n.d.; Groen *et al*., n.d.; de Vos *et al*., 2017). Moreover, a simulation study showed that networks with more strongly connected symptoms showed transitions to a depressed state more often compared with networks with weak connections (Cramer *et al*., 2016). Furthermore, exposure to external stress resulted in sudden shifts in symptoms only in strongly connected networks. Within such networks, removal of the stressor after the phase transition occurred did not cause the system to shift back to its original state (Cramer *et al*.). The fact that recovery is not linearly related to the removal of the cause of the shift is called ‘hysteresis’. Such non-linearity is typical for complex systems. This phenomenon also has face validity for the field of psychiatry as it may explain why people remain stuck in a clinical state even after removal of certain provocative factors that had prompted the mental complaints.

Finally, the most direct support for the idea that symptom changes behave according to the principles observed in the complex system stems from empirical research showing that transitions in symptom levels can be anticipated by directly assessing changes in the stability of the system. From other fields of study (e.g. ecology and computer science), it is known that these changes in stability can be observed using certain ‘EWS’ (Tretyakov *et al*., 1998; van Nes and Scheffer, 2007; Dakos *et al*., 2008). Such signals involve changes in the dynamics of important variables of a complex system, like increasing levels of autocorrelation (i.e. the current state of an element of the system becomes a better predictor for its future state), variance (i.e. elements of the system show greater amplitude changes in their intensity levels) or flickering (sudden changes in intensity levels). These reflect increasing instability of the system and have been shown to closely precede critical phase transitions in various sorts of complex systems. Considering the above, we expect that EWS (which involve more complex aspects of time-series dynamics than simple intensity changes of symptoms) may signal an increased likelihood of a phase transition and that such EWS can predict such transitions substantially earlier than simple changes in mean levels of these symptoms. This means that if psychopathology also behaves as a complex system, we may be able to find EWS that we can use to foresee important shifts in symptoms in an earlier phase and in a personalized manner. A few recent empirical studies already found support for this hypothesis (Schiepek *et al*., 2009; van de Leemput *et al*., 2014; Wichers *et al*., 2016). For example, in the time-series experiment in which a patient was followed over 239 days completing multiple measurements of mental states a day, EWS were observed in the sum score of all measured mental states. These EWS anticipated a subsequent phase transition in depressive symptoms (Wichers *et al*., 2016). Although these findings still await replication by a large-scale study in which EWS are followed over time within persons, support for the idea that psychopathology behaves as a complex system is accumulating. To conclude, many hypotheses have been formulated for the application of complex systems theory for the field of psychiatry and some empirical studies have been carried out suggesting we may be able to foresee transitions in levels of symptoms based on the system's changes in stability. We now want to further explore what this theoretical framework can do for foreseeing the *type of symptom shifts* that individuals may express in the near future. Before we move to the above mentioned theoretical explorations, however, it is important to first discuss the nature of psychopathology.

### Redefining psychopathology as a multidimensional space

The idea that mental disorders are distinct and independent entities is not supported by empirical evidence (Carragher *et al*., 2015). All evidence points to the fact that diagnostic classifications are not independent and show huge overlap with one another (Kessler *et al*., 2005; Merikangas *et al*., 2010). Comorbidity is a rule rather than exception (Krueger and Eaton, 2015). Also, patterns of symptoms within diagnoses are quite heterogeneous (Wardenaar and de Jonge, 2013). The human-made top-down boundaries and classifications established in diagnostic manuals may thus distort our view on the real structure of psychopathology. Clear boundaries between mental disorders seem absent and a patient's pattern of symptoms seems to spread across the various dimensions of psychopathology to form unique clusters of problems in each person. Despite such continuity, however, empirical studies show that some types of symptoms lump together more often while other combinations of symptoms hardly ever co-occur (Goekoop and Goekoop, 2014; Boschloo *et al*., 2015; Bekhuis *et al*., 2016). A common explanation for the co-occurrence of certain symptoms or mental states (e.g. feeling hopeless, guilty, down, fatigued, irritated) is that an underlying latent entity is responsible (e.g. depression). However, this view alone cannot explain that the precise combinations of symptoms differ between people and can be mixed with symptoms from other clusters. A more recent view is the network perspective, which does not necessarily assume an underlying causal entity, but theorizes that symptoms can also trigger each other and thereby form clusters of co-occurring symptoms in a self-organized, or bottom-up fashion (Cramer *et al*., 2010; Goekoop and Goekoop, 2014; Borsboom, 2017). We can then imagine a reality in which no absolute boundaries exist between diagnoses, and in which symptoms of psychopathology are all, to a varying extent, related to each other within one psychopathological space. However, some types of symptoms or mental states seem to lump together more often in this space while other combinations of symptoms hardly ever co-occur. From the network perspective, this may make sense, as it can be easily imagined that one mental state, for example, ‘feeling down’ easily triggers a certain other mental state, like ‘worrying’, but not so easily the mental state representing the feeling of being watched. Thus, certain mental states may be further apart from each other in space than other mental states. Similarly, certain groups of mental states may all be at a relatively close distance – meaning that they easily activate each other – making it likely that they are eventually ‘switched on’ together. Such groups may represent combinations of symptoms that have been frequently observed together and therefore received a group-identifying label such as ‘depression’ or ‘psychosis’.

**Fig. 2.**
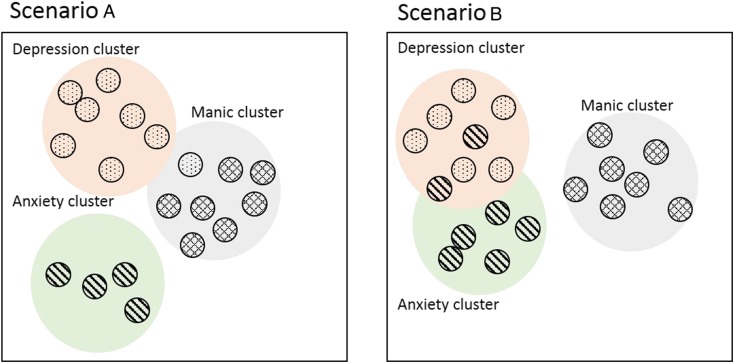
Multidimensional space of psychopathology symptoms. The circles represent different symptoms and the distance between them represents the ease with which they can trigger their neighbour symptom. The coloured groups represent different clusters of symptoms. In scenario A, it is more likely that depressive symptoms may eventually trigger the manic cluster than in scenario B, while depressive symptoms in scenario B will more easily activate anxiety symptoms.

Thus, symptoms and dimensions of psychopathology show preferential connections, which put constraints on the likelihood of certain psychopathological syndromes. Despite such global constrains, however, individual patients are known to differ in the wiring patterns of their psychopathology networks, which give rise to unique deviations from group-level connectivity. Thus, whereas in one person depressed feelings (such as sadness, guilt or shame) can easily trigger anxiety symptoms (such as worrying or inner tension) because they are close in the multidimensional space, this is not necessarily true in another person (see Fig. 2). Second, not only the connections between the clusters (lumps) of symptoms, but also the clusters themselves are likely to differ per individual with regard to their precise content: whereas for one person anxiety and depressive symptoms may form a single cluster or lump, this may not necessarily be the case for the next person. This implies that the structure of psychopathology and its supposed dimensions may differ per person and may not even be stable over time within a person. These theoretical ideas on the nature of psychopathology may explain the observed heterogeneity in symptom profiles (see Fig. 2).

## Applying complex systems theory to foreseeing specific types of symptom transitions

**Fig. 3.**
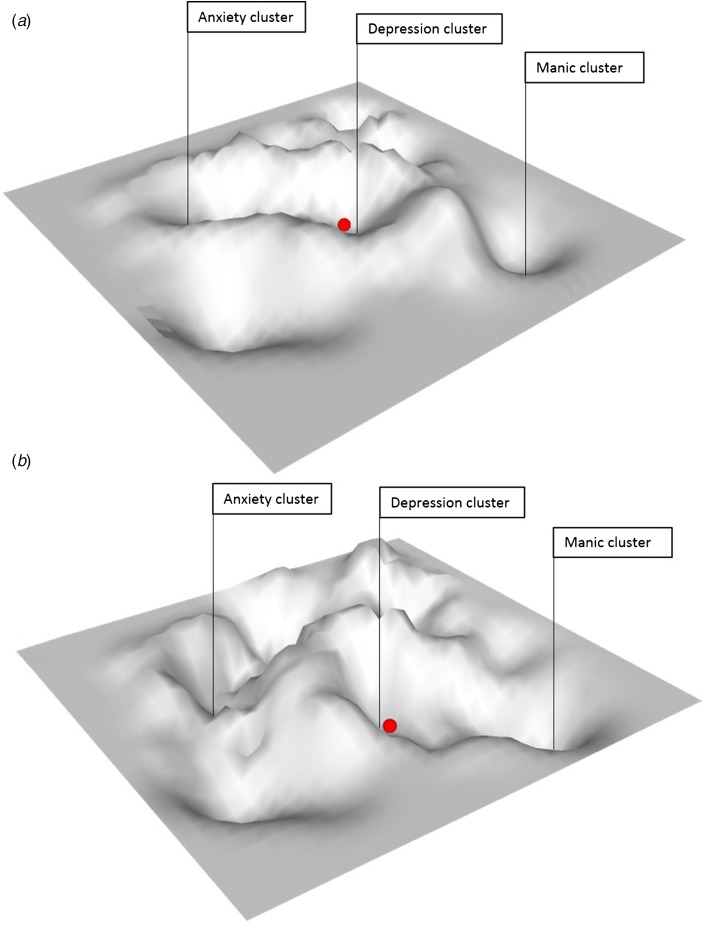
Three dimensional stability landscape. Landscape (a) shows a situation in which an individual in a currently depressed state is close to the basin of attraction towards developing anxiety symptoms but not, for example, to developing manic symptoms. In landscape (b), the situation is reversed; this individual who is currently in a depressed state is close to the attractor towards developing manic symptoms but not towards developing anxiety symptoms.

As mentioned previously, the possibility to differentiate what type of symptom transitions is likely to occur in vulnerable individuals is highly relevant. The question is whether generic principles that apply to complex systems are informative also on this matter. If symptoms all relate to each other within a multidimensional space as explained above, and if there are only relative boundaries between symptoms and symptom clusters, then instable attributes of the system at large may in theory inform us on the risk of transitions across the whole of psychopathology. This would mean that rather than working with a two-dimensional (2D) stability landscape (see Fig. 1), we suggest a 3D stability landscape (Fig. 3) in which system stability is depicted not only on the dimension from low to high stability for psychopathology in general, but also for different dimensions of psychopathology. In this 3D landscape, we could then observe that the state of the system of an individual, who is, for example, currently experiencing depressive symptoms, is close to a basin of attraction towards developing anxiety symptoms but not, for example, to developing manic symptoms (see Fig. 3a). However, in the 3D landscape of another person, this could be precisely opposite (Fig. 3b).

We know that the chances of individuals to develop various forms of psychopathology differ from one person to another. Therefore, it seems logical to expect that the same principles of system stability play a role in signalling phase transitions to a certain set of symptoms (e.g. manic symptoms), rather than to another (e.g. anxiety symptoms), depending on one's individual settings in the multidimensional space. The probability of making a transition to a certain group of symptoms is then reflected in the instability of a system's current state to move towards the corresponding basin of attraction of that particular group of symptoms in the psychopathological space. In a 3D stability landscape, we may assume that this stability is not equal around all basins of attraction. For example, at some point in time, the state's position within the stability landscape can make it very easy for the state to roll into a large basin of attraction corresponding to anxiety symptoms (i.e. move along the anxiety dimension), while it will not likely roll into the basin corresponding to manic symptoms (i.e. move along the dimension of mania; see Fig. 3a).

### EWS and local points of instability

**Fig. 4.**
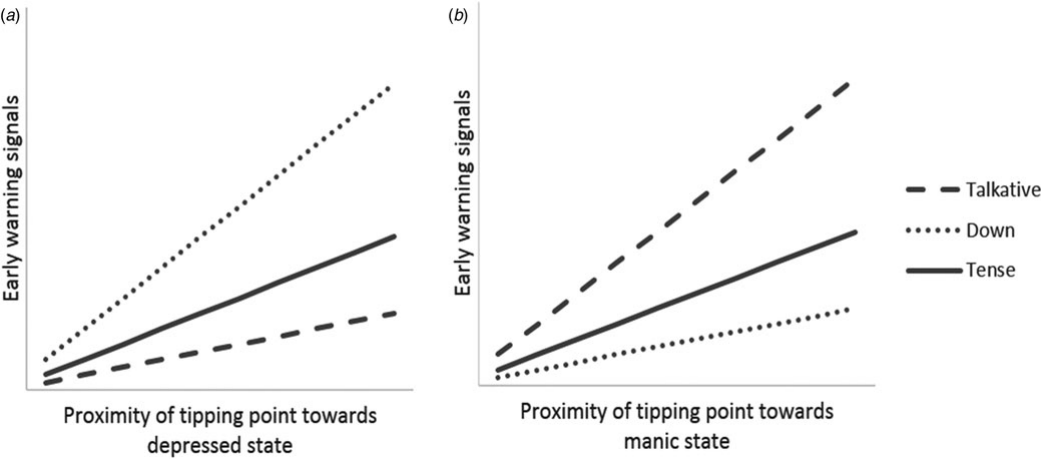
Association between early warning signals (EWS) and the proximity of tipping points. As a system approaches a tipping point, a transition towards an alternate state becomes more likely. (a) A system that is likely to shift towards a depressed state is hypothesized to show pronounced EWS in related mental states, such as feeling down. (b) In contrast, future transitions towards manic states might be preceded by EWS in other mental states, such as feeling talkative. Patterns in EWS might therefore reflect the direction of future transitions.

The above suggests the possibility that ‘local points of instability’ exist in the landscape that are related to specific symptom transitions. For example, we can assume that if we find rising EWS specifically, for example, in patterns of the mental state ‘feeling down’, that this is more likely to signal a transition towards depressed states than towards other symptom clusters. Similarly, rising EWS in other mental states, for example, ‘feeling tense’ or ‘being extremely talkative’, may be less pronounced prior to transitions towards depression relative to transitions towards anxious or manic states, respectively (Fig. 4). A first novel assumption therefore is the idea that EWS, such as rising autocorrelation or variance, may signal local points of instability in the 3D psychopathology landscape and can therefore predict what type of symptom transitions are most likely to occur. If this assumption is correct, then EWS patterns of various mental states may inform us not only on the possibility of a nearby transition, but also on the type of psychopathology that may develop in the near future.

### Network structure and local points of instability

**Fig. 5.**
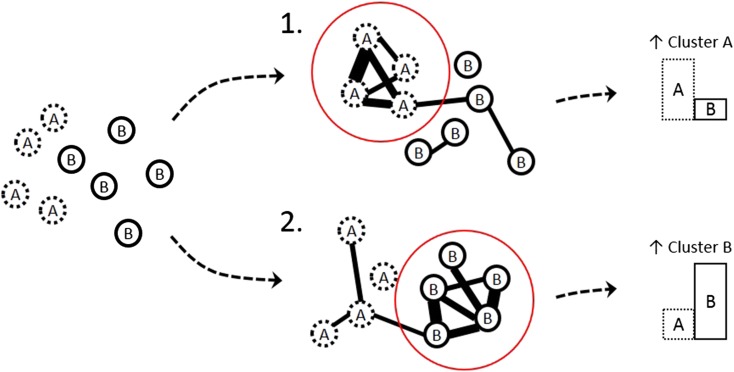
Network connectivity as a prognostic indicator. Reported symptoms are a mix of clusters A and B. The dynamic networks reveal two different scenarios. In scenario 1, the dynamic network shows high connectivity in cluster A symptoms, while in scenario 2, the dynamic network shows high connectivity in cluster B symptoms. The network theory predicts that this leads to different likelihood of future symptomatology. Scenario 1 signals an increased likelihood to further develop symptoms of cluster A, while scenario 2 signals an increased likelihood to further develop symptoms of cluster B.

A second hypothesis that may follow from the above described multidimensional view on psychopathology is that *the structure of connections* between symptoms may also inform us on the likelihood of specific directions of symptom transitions. As explained above, symptoms are expected to trigger other symptoms, leading to clusters of symptoms that we label as syndromes (e.g. depression, anxiety, psychosis). If no absolute boundaries exist then we can expect that symptoms can connect not only within fixed groups of symptoms but also across such groups. Symptoms that connect across boundaries are called ‘bridge symptoms’. These ideas have been formulated previously (Cramer *et al*., 2010; Goekoop and Goekoop, 2014) and can explain why people with one categorical psychiatric diagnosis (e.g. unipolar depression, when a depression cluster is active) are more likely to later fulfil the criteria for another diagnosis (e.g. schizoaffective disorder, when a psychosis component joins in). Since bridge symptoms facilitate most of the communication between the clusters (syndromes), changes in the states of these bridge symptoms may be particularly good candidates for the prediction of the direction of phase transitions in psychopathology networks. What follows from these ideas is that the specific patterns of connections between symptoms, like the EWS, may provide us with clues regarding the likelihood of a transition to a particular set of symptoms (a dimension in the landscape) (Fig. 5). For instance, if feeling down strongly triggers being paranoid in a particular individual, and if being paranoid is strongly connected to (and easily activates) many other symptoms that make up the cluster of ‘psychotic symptoms’, then we can say that the state of feeling down is proximal and close to the transition towards psychosis for this person. In the 3D stability landscape, this situation would be reflected by a gully in the hilly landscape running from the state of feeling down towards the basin of attraction corresponding to a psychotic state. In such a way, the network structure of an individual's symptoms may be informative for local points of instability in the multidimensional space of psychopathology and may signal, depending on the current activation state of the network, what direction of transition is most likely. We thus hypothesize that both approaches, EWS and network structure, may inform on the direction of symptom transitions. An interesting question is whether both approaches are complementary in providing information and whether the combination of information further increases the precision of the estimated likelihood. This question needs to be resolved empirically.

## Operationalization in novel research designs and clinical implications

Above we have tried to explain how a complex systems view may lead to assumptions concerning the direction of symptom shifts. It is important, however, to not only create new theories on the nature of psychopathology, but also to pave the way for empirical verification of the proposed ideas. This requires a translation from the concept of system instability to the direct testing thereof, formulating specific predictions and utilizing novel research designs. We will discuss each of these challenges. The concept of system instability is often used as a nice metaphor. However, recent discoveries by Scheffer *et al.* (Scheffer *et al*., 2009, 2012), showing that system instability can be estimated by specific EWS, make system instability empirically testable. Whereas time-series of a CO_2_ proxy were used to find EWS on climate change (Scheffer *et al*., 2009), time-series of emotions or mental states can be used to estimate EWS anticipating symptom transitions. Experience sampling, a diary technique to sample people's experiences in the flow of daily life (Myin-Germeys *et al*., 2009), is one possibility to get access to such information. An operationalization hereof has been described by Wichers *et al.* (Wichers *et al*., 2016). Similar statistical models as used in this study to estimate system instability with EWS can be used to test the currently proposed novel hypotheses regarding predictions on the precise type of symptoms that are likely to make a transition (i.e. via local points of instability visualized in a 3D rather than a 2D stability landscape). However, in a 3D stability landscape, the situation is a bit more complex. For example, to derive an approximation of the stability landscapes as depicted in Fig. 3, one needs to examine EWS patterns in multiple mental states in these depressed individuals. Strongly rising levels in EWS patterns in certain mental states (e.g. in anxiety) would then correspond with instable locations in the landscape where the state of the system can easily move towards that specific attractor (in that case the anxiety attractor). In that way, (multiple) local instabilities in a system become empirically testable.

However, these empirical tests have their statistical and methodological complexities. For example, the current research questions explicitly hypothesize changing EWS patterns over time. However, most statistical models assume stationarity. Fortunately, there are some solutions available. A first solution, also used in the *n* = 1 experiment (Wichers *et al*., 2016), is to make use of moving window techniques. This means that autocorrelation, as one form of EWS, is estimated separately in a high number of overlapping time windows that in total cover the complete time period of investigation. Another solution is to use the recently developed time-varying vector autoregressive models (Bringmann *et al*., 2017) that allow for changing parameters over time.

Another complexity in the empirical testing of these ideas is that they require intensive measurement regimes, which should be timed in a period during which transitions are likely to occur. Also, to derive an approximation of the stability landscapes as depicted in Fig. 3, one needs to examine EWS in a broad range of mental states (i.e. a depression cluster and its neighbouring clusters, such as anxiety, mania and inhibition clusters). This requires the use of highly parsimonious yet informative questionnaires and data collection techniques. A question is whether such intensive designs are a feasible option in (high-risk) psychiatric populations. Recently, however, intensive time-series datasets (with a length of 3–6 months and with a sampling frequency of once to five times a day) have been successfully collected in patient groups with severe psychiatric problems. Compliance on measurements reached 76% on average and focus groups revealed that patients felt positive regarding the idea of monitoring themselves in such a way as it helps them to acquire more insight into their symptoms (Bos *et al*., n.d.; Schiepek *et al*., 2016). Thus, although data collection is intensive, application hereof in clinical groups, and with the promise of using it for improving personalized prediction of course, may be well feasible. Nevertheless, we need to keep in mind that using complex system tools for this purpose only has added benefit if these outweigh more simple measures, such as plain mean levels of mental states or patients’ own indications of risk, to predict symptom trajectories in psychopathology.

In short, psychiatry is in need of personalized approaches. If we succeed, this novel approach may yield strongly improved personalized estimations on the course of psychiatric symptoms. Such information can be extremely useful for early intervention strategies aimed at preventing specific psychiatric problems.
